# Role of tau N-terminal motif in the secretion of human tau by End Binding proteins

**DOI:** 10.1371/journal.pone.0210864

**Published:** 2019-01-22

**Authors:** C. Laura Sayas, Miguel Medina, Raquel Cuadros, Ivanna Ollá, Esther García, Mar Pérez, Isidro Ferrer, Félix Hernández, Jesús Avila

**Affiliations:** 1 Centre for Biomedical Research of the Canary Islands (CIBICAN), Institute for Biomedical Technologies (ITB), University of La Laguna (ULL), Tenerife, Spain; 2 Network Center for Biomedical Research in Neurodegenerative Diseases (CIBERNED), Madrid, Spain, CIEN Foundation, Queen Sofia Foundation Alzheimer Center, Madrid, Spain; 3 Centro de Biología Molecular Severo Ochoa (CBMSO) CSIC-UAM, Madrid, Spain; 4 Departamento de Anatomía Histología y Neurociencia, Facultad de Medicina UAM, Madrid, Spain; 5 Department of Pathology and Experimental Therapeutics, University of Barcelona, Barcelona, Spain; 6 Service of Pathologic Anatomy, Bellvitge University Hospital, Barcelona, Spain; 7 Institute of Neurosciences, University of Barcelona, Hospitalet de Llobregat, Spain; McGill University, CANADA

## Abstract

For unknown reasons, humans appear to be particular susceptible to developing tau pathology leading to neurodegeneration. Transgenic mice are still undoubtedly the most popular and extensively used animal models for studying Alzheimer’s disease and other tauopathies. While these murine models generally overexpress human tau in the mouse brain or specific brain regions, there are differences between endogenous mouse tau and human tau protein. Among them, a main difference between human and mouse tau is the presence of a short motif spanning residues 18 to 28 in the human tau protein that is missing in murine tau, and which could be at least partially responsible for that different susceptibility across species. Here we report novel data using affinity chromatography analysis indicating that the sequence containing human tau residues 18 to 28 acts a binding motif for End Binding proteins and that this interaction could facilitate tau secretion to the extracellular space.

## Introduction

In different tauopathies, including Alzheimer’s disease (AD), it has been proposed that tau pathology takes place through the spreading of toxic extracellular tau species between anatomically connected brain regions [1]. By using mouse models expressing different forms of human tau, it has been found that tau pathology spreading in the brain of those mice mainly occurs through the human rather than the endogenous mouse tau molecules [2, 3]. However, this point should be further studied [4, 5].

“Prion-like” propagation of tau pathology from neuron to neuron has been proposed to involve several steps, including secretion of tau to the extracellular space [6]. Recent evidence has shown that in physiological conditions tau can be secreted and found extracellularly in the absence of cell death [7]. This extracellular tau needs then to contact with a neighboring neuron to propagate its toxic effect [8, 9]. Humans appears to be particularly vulnerable to tau-dependent neurodegeneration. It has been in fact postulated that human and mouse tau could present some differences in their capacity for pathology propagation, one of which could be a distinct capability to be secreted.

Since there is usually a close relationship between structure and function and human and mouse tau slightly differ in their primary structure, we have investigated whether a specific human tau sequence exists that could better facilitate its secretion outside the cell. In fact, the most striking difference between human and mouse tau molecules is the sequence spanning residues 18 to 28, a motif which is present in human tau but absent from mouse tau [10, 11].

In order to analyze whether this motif might affect the secretion process, we have transfected wild type human tau (htau from now on) or human-“murinized” tau lacking residues 18 to 28 (from now on, htau-**Δ**18–28) in non-neuronal cells and studied the extracellular levels of the different tau species. Our results show a preferential secretion of human tau molecule bearing residues 18 to 28. In addition, by using a synthetic tau peptide containing residues 16 to 28 of human tau, we have found that it binds to End Binding (EB) proteins, which belong to the group of microtubule plus-end tracking proteins (+TIPs) [12]. Indeed, we had previously shown that tau interacts with EBs in neuronal cells, regulating their localization and function [13]. Since EB proteins have been involved in the secretion of some other proteins [14], we analyzed their potential participation in tau secretion. Our data indicate that overexpression of EBs increase human tau secretion whereas EBs downregulation leads to a reduction in the release of human tau to the extracellular space. Thus, we propose a working model in which the human tau motif 18 to 28 could facilitate tau secretion through a mechanism involving EB proteins.

## Materials

Synthetic human tau peptide GTYGLGDRKDQGG containing residues 16 to 28 was obtained from ABYNTEK BIOPHARMA S.L. (Bizkaia, Spain). Human tau peptide (tau 3R) containing repeats 1,3 and 4 was isolated as previously described [15]. Brain extracts were obtained from autopsies on non-demented controls performed at the Hospital of Bellvitge (S1 Table; Barcelona, Spain). Human Brian samples were approved by the Ethic Committee of Bellvitge Hospital, Barcelona, Spain. All ethical-legal documents of the brain bank, including written informed consent, were approved by an ethics committee external to the bank. Human cDNA tau was obtained as previously reported [15]. EB1 antibody was from BD Transduction Laboratories (#610535) and EB3 antibody was from Abcam (#157217). Tau rabbit polyclonal antibody was from Novusbio (NB100-82247). β-tubulin (clone tub 2.1.T4026) and β-actin were obtained from Sigma (A-5441).

## Methods

### Cell culture

Fibroblast-like monkey COS-7 cells (ATCC, Rockville, MD, USA) were routinely grown in Dulbecco’s modified Eagle’s medium (DMEM), containing 10% fetal bovine serum (FBS), 2 mM of L-glutamine, 100 U/mL penicillin, and 100 mg/mL streptomycin. HEK-293-tau3R, a stable cell line overexpressing human tau generated in our laboratory [16] were grown in DMEM supplemented by 10% USA FBS, 2 mM of L-glutamine, 100 U/mL penicillin, and 100 mg/mL streptomycin, and the selection antibiotic zeocin (200μg/ml). Cultures were maintained at 37 °C in a humidified atmosphere containing 5% CO2. [17]

### Affinity chromatography

Human tau peptide (residues 16 to 28), or human tau fragment containing 1, 3 and 4 repeats (T3R) were covalently linked to a Sepharose 4B column. On that column, a human brain cell extract was chromatographed in the conditions previously indicated [18], and the bound proteins were characterized by Western blot or dot blot analysis.

### Plasmids and transfection

Wild-type human Tau was expressed from the SV40 early promoter using the plasmid pSGT42 previously described that express the longest human Tau isoform present in the Central Nervous system [19]. Synbio Tech (NJ, US) engineered htau42-**Δ**18–28 using pUC57 plasmid. Then, pUC57- htau42-**Δ**18–28 was digested with EcoRI and BglII. The smaller 1.3 Kb fragment was purified and ligated into the biggest fragment from pSGT42 expressing human Tau previously digested with EcoRI and BglII to obtain pSGhTau42-Δ18–28. Positive clones were analyzed by restriction analysis to test for the proper orientation and correct size of the inserts. Finally, the constructions were confirmed by DNA sequencing analysis.

HEK-293-tau3R cells were transfected with EB1-GFP or EB3-GFP constructs (a generous gift of Dr. N. Galjart [20, 21]) using LipofectamineTM 2000 (Invitrogen, Carlsbad, CA, USA), following the manufacturers’ protocol. Cells were harvested 48 h post-transfection.

### shRNA plasmids and lentiviral transduction

The effect of different human EB1 shRNA lentiviral vectors (Mission; Sigma Aldrich, St. Louis, MO, USA) was checked by transduction in HEK-293-tau3R cells. The most effective one in down-regulating endogenous EB1 was selected and used (SHCLND-NM_012325 (TRCN0000062142): 5´-CCGGGTTCAGTGGTTCAAGAAGTTTCTCGAGAAACTTCTTGAACCACTGAACTTTTTG-3´). A plasmid bearing a scrambled (non-targeting) shRNA sequence was used as a control. Recombinant lentiviral particles were obtained by co-transfection of sub-confluent HEK-293T cells using Lipofectamine 2000 (Invitrogen, Carlsbad, CA, USA), with the EB1 shRNA construct and both pCMVdR8.74 (Addgene, Cambridge, MA, USA) and pMD2G (Addgene, Cambridge, MA, USA) plasmids. Lentiviruses were collected 48 h post-transfection. HEK293-tau3R cells were infected either with the scrambled shRNA virus (control) or with the EB1 shRNA virus. Cells were harvested 72 hours post transduction for further experiments.

### Tau secretion assays

Tau release was measured in conditioned medium of HEK-293-tau3R or COS-7 cells, as described previously [13]. In brief, the culture media was collected either 48 h post-transfection or 72 h post lentiviral transduction, and first centrifuged at 300xg for 10 min. The supernatant was then subjected to centrifugation at 2000xg for 10 min, and the new supernatant was centrifuged once again at 10000xg for 30 min at 4 °C to remove cell debris. The obtained supernatant, designated as “Final supernatant”, was then boiled and subject to SDS-PAGE and Western blot analysis.

### Cell lysates and western blotting

Cells were harvested -after transfection or lentiviral transduction- in cold lysis buffer containing: 20 mM HEPES pH 7.4, 5 mM EDTA, 100mM NaCl, 1% Triton X-100, 0.1mM sodium orthovanadate, 1x anti-protease cocktail and 1 μM okadaic acid. Lysates were centrifuged at 10000xg for 10 minutes at 4 °C and boiled in Laemmli’s buffer. Protein samples concentrations were quantified by the BCA protein assay. 15–20 μg of each cell extract or equal amounts of “total supernatants” were run on 10% SDS-PAGE gels and electrophoretically transferred to nitrocellulose membranes (Schleicher & Schuell, Gmbh, Munich, Germany). The filters were blocked with 5% semi-fat milk powder in phosphate-buffered saline (PBS)-0.1% Tween 20 (PBS-T), and subsequently incubated with primary antibodies overnight (at 4 °C). After three washes, the membranes were incubated with the corresponding peroxidase-conjugated secondary antibody (DAKO, Carpinteria, CA) for 1 h (DAKO), and then washed three times again in PBS-Tween 20. Immunoreactivity was visualized by Western Lightning reagents (Perking Elmer Life Science, Whaltam, MA, USA).

### Data analysis

Data are presented as the mean ± SD (n = 2–3). Two group comparisons were made using unpaired Student’s two-tailed t-test. Significance was accepted at p < 0.05.

## Results

### Generation and expression of htau-Δ18–28

**Fig 1:**
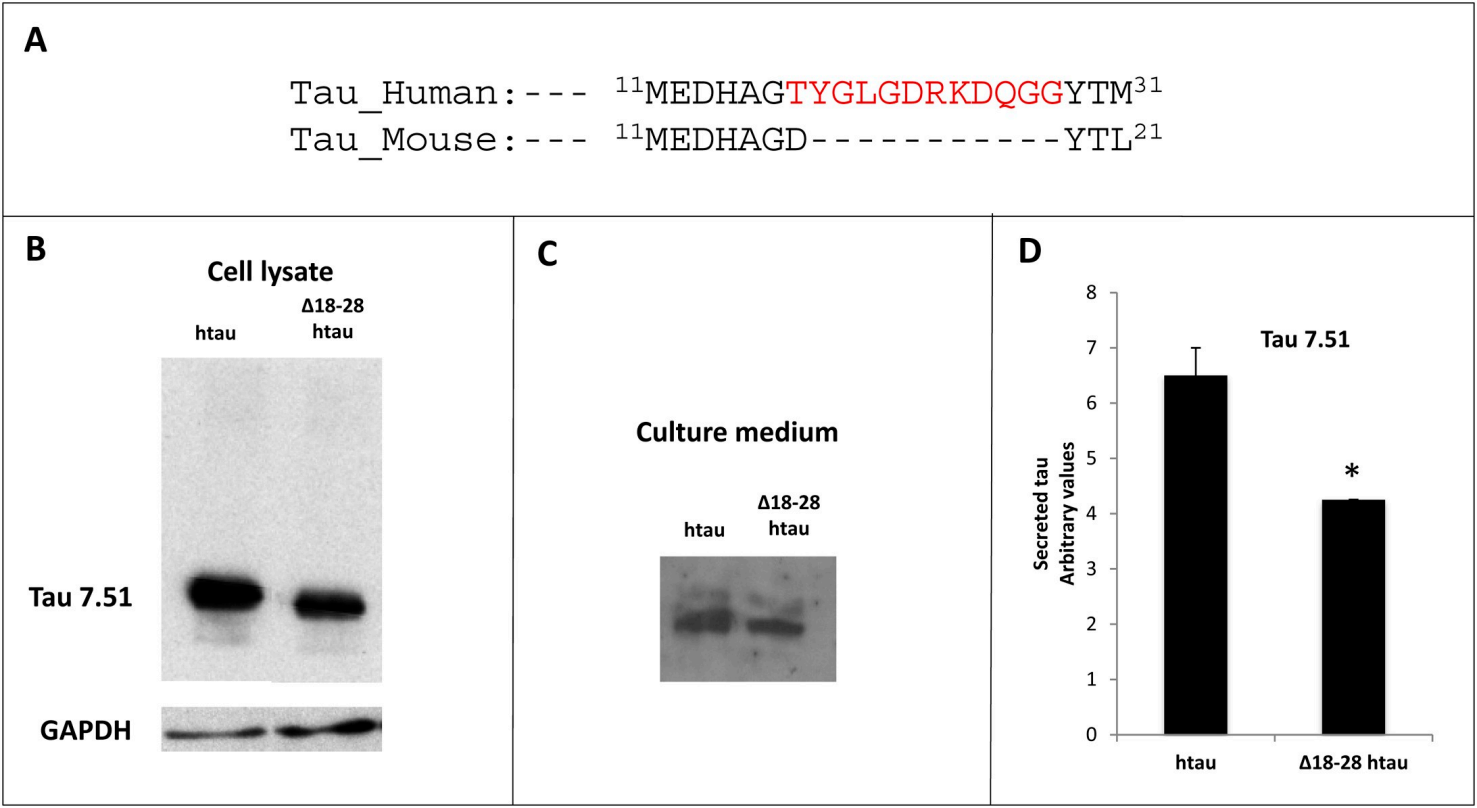
Expression of human and human tau-Δ18–28 in COS-7 cells. A) Alignment of human and mouse tau sequences at the N-terminal domain. Mouse tau lacks the motif spanning residues 18–28 that is present in human tau. B) Expression of human and htau-**Δ**18–28 in COS-7 cells. Tau expression was analyzed by Western blot using tau antibody 7.51. GADPH was used as loading control for cell lysates. C) Secreted tau levels from experiment 1B were analyzed by Western blot of cell culture medium. D) Densitometric quantitation of the extracellular tau from panel C. *P < 0.05.

The most striking difference between the primary structures of human and mouse tau is the presence in the human sequence, but not in mouse tau, of a motif spanning residues 18 to 28 (Fig 1A). To assess the possible functional effects of bearing this amino acid sequence, this 18–28 motif was removed from a human tau cDNA construct. Human “murinized” tau (htau-**Δ**18–28) or human tau (htau) were expressed in COS-7 cells and their expression was analyzed by Western blot (Fig 1B). htau-**Δ**18–28 shows a slightly higher electrophoretic mobility than htau, probably as a consequence of the smaller size of htau-**Δ**18–28 as compared to that of full length htau. Similar results were observed when the expression of both constructs was tested in other non-neuronal cell lines such as HEK-293T cells (not shown). These data indicate that htau-**Δ**18–28 can be expressed in non-neuronal cells, resulting in a protein with a slightly smaller molecular weight as compared with htau.

### Htau-Δ18–28 is less efficiently secreted than htau upon overexpression

We then analyzed whether removal of residues 18 to 28 had any effect on the tau secretion process. We found that when similar amounts of tau proteins were overexpressed in non-neuronal cell cultures (Fig 1B), the levels of secreted full length htau were significantly higher than those observed with htau-**Δ**18–28 (Fig 1C and 1D). Such increased levels of extracellular htau was not the consequence of differences in cell death upon transfection with both cDNAs, which were similar in both cases (data not shown). Our data point to a reduction of tau secretion upon removal of the 18–28 amino acid sequence.

### Human tau peptide 16–28 binds to eb proteins

In order to look for for proteins that could bind specifically to this motif and perhaps regulate tau secretion, a peptide spanning residues 16–28 of the human tau sequence was coupled to a Sepharose column and an affinity chromatography analysis was performed by passing a human brain extract over the column.

**Fig 2:**
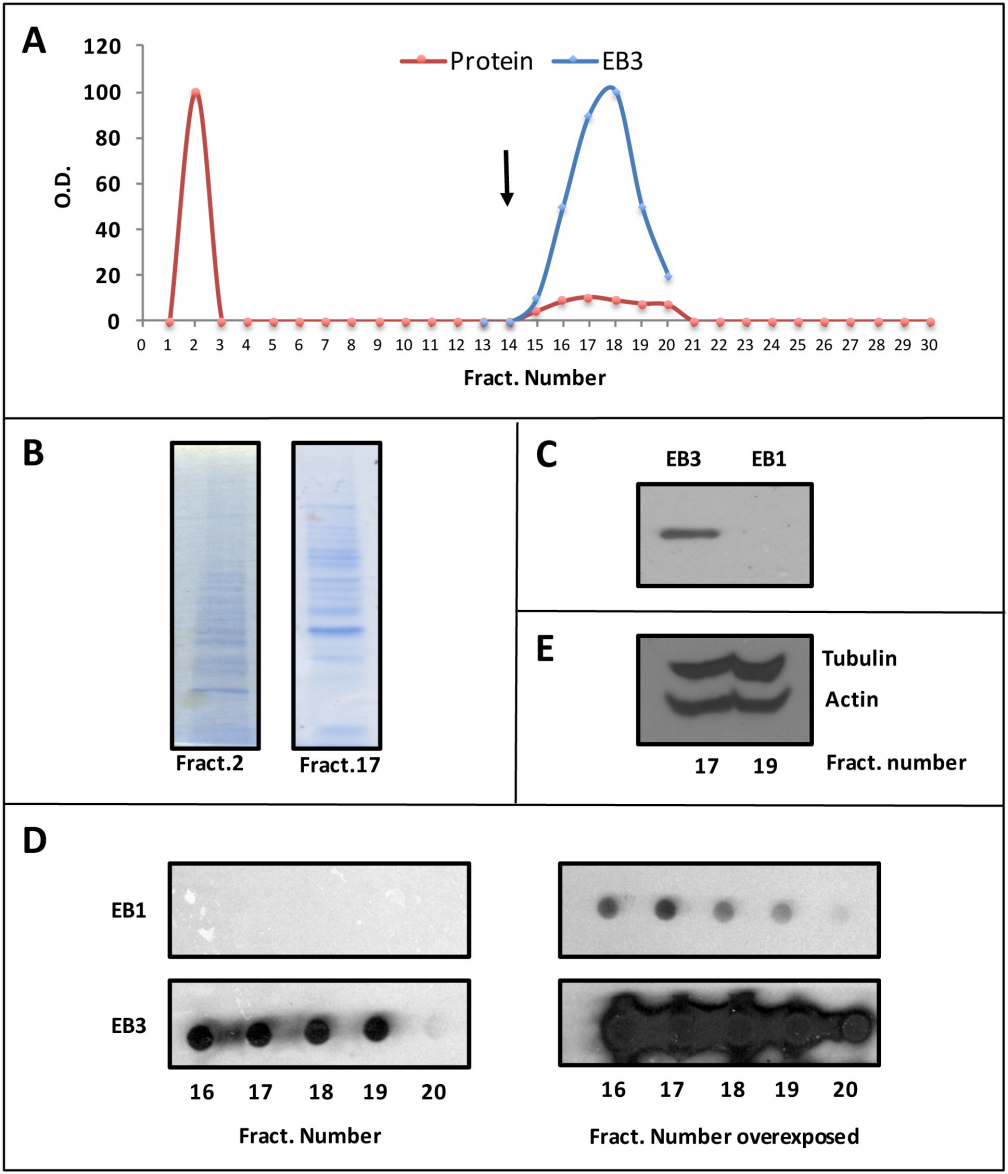
Binding of EB3 protein to human tau peptide 16–28. A) A human brain cell extract was chromatographed on a Sepharose column containing covalently linked a human tau peptide spanning residues 16–28. The cell extract proteins bound to the column were eluted by salt addition (see arrow). B) The unbound (fraction 2) and bound (fraction 17) proteins were characterized by gel electrophoresis. C) Western blot using specific antibodies against EB3 or EB1 proteins. D) Exposed and overexposed dot blots to analyze the presence of EB1 or EB3 proteins in the indicated eluted fractions. E) Western blot analysis using a mix of actin and tubulin antibodies tested on fractions 17 and 19.

Fig 2 shows that upon chromatography, some human brain proteins were eluted from the column by increasing salt concentration in the chromatography buffer. Since EB proteins have been previously shown to bind tau in neuronal cells and brain tissue [13, 22] as well as being involved in the secretion of some proteins [14], we analyzed by Western blot the presence of those proteins in the human tau peptide-bound fractions. By using specific antibodies reacting with EB1 or EB3 we found that mainly EB3 protein was among those bound to tau peptide (Fig 2A), while EB1 was not detected. Similar results were obtained by dot blot, with EB3 being present in large amounts and EB1 undetected (Fig 2B and 2C although when the blots were overexposed, a small amount of EB1 was also found to be present (Fig 2D), indicating that EB1 is also associated with the tau 16–28 peptide. These data show that both EB1 and EB3 are able to bind to the human tau residues 16 to 28, suggesting that EB proteins might be involved in human tau secretion. As indicated below, the smaller amount of EB1 bound to tau peptide could be the consequence of the very low levels of EB1 present in the human brain cell extracts compared to the EB3 levels.

### Eb binding sites within tau molecule

**Fig 3:**
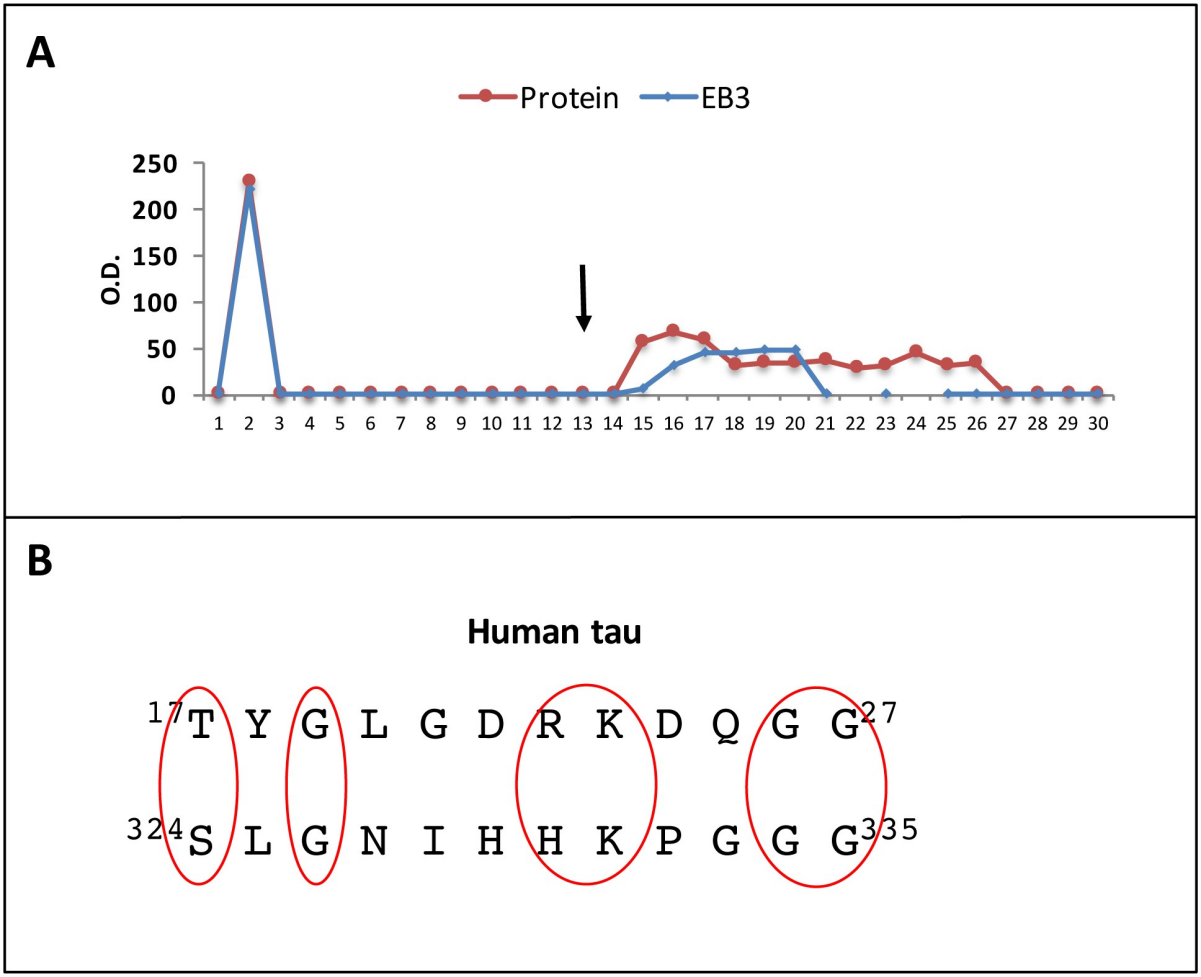
Binding of EB3 protein to a tau peptide containing microtubule binding repeats 1, 3 and 4. A) Tau 3R fragment containing tau repeats 1, 3, and 4 was covalently linked to a Sepaharose column and a human brain cell extract was chromatographed on that column, in a similar fashion to that indicated in Fig 3. The dot blot analysis indicated the presence of EB3 in the bound protein fractions. B) The similarity of human tau sequences spanning residues 17–27 and 324–335 (within the microtubule binding domain) is shown.

As mentioned above, it has recently been shown that EB proteins recognize the microtubule-binding repeats of human tau *in vitro* [22]. In Fig 3A, we confirmed this binding by affinity chromatography, since EB3 interacts with a tau peptide bearing three microtubule-binding repeats (1, 3 and 4) coupled to a Sepharose column. This result suggests that microtubule-binding repeats and human tau peptide 16–28 might have some sequence similarities, as confirmed in the sequence alignment presented in Fig 3B, that could explain the binding to EB3 in both regions. On the other hand, we found that both tau peptides, the one containing 3 microtubule binding repeats (not shown) and the one with residues16-28 (Fig 2C) can bind to actin and tubulin. These data indicate that human tau protein might present different binding sites for EB proteins, including at least the microtubule binding domain and the 16–28 motif.

### Overexpression of EB proteins facilitates tau secretion

**Fig 4:**
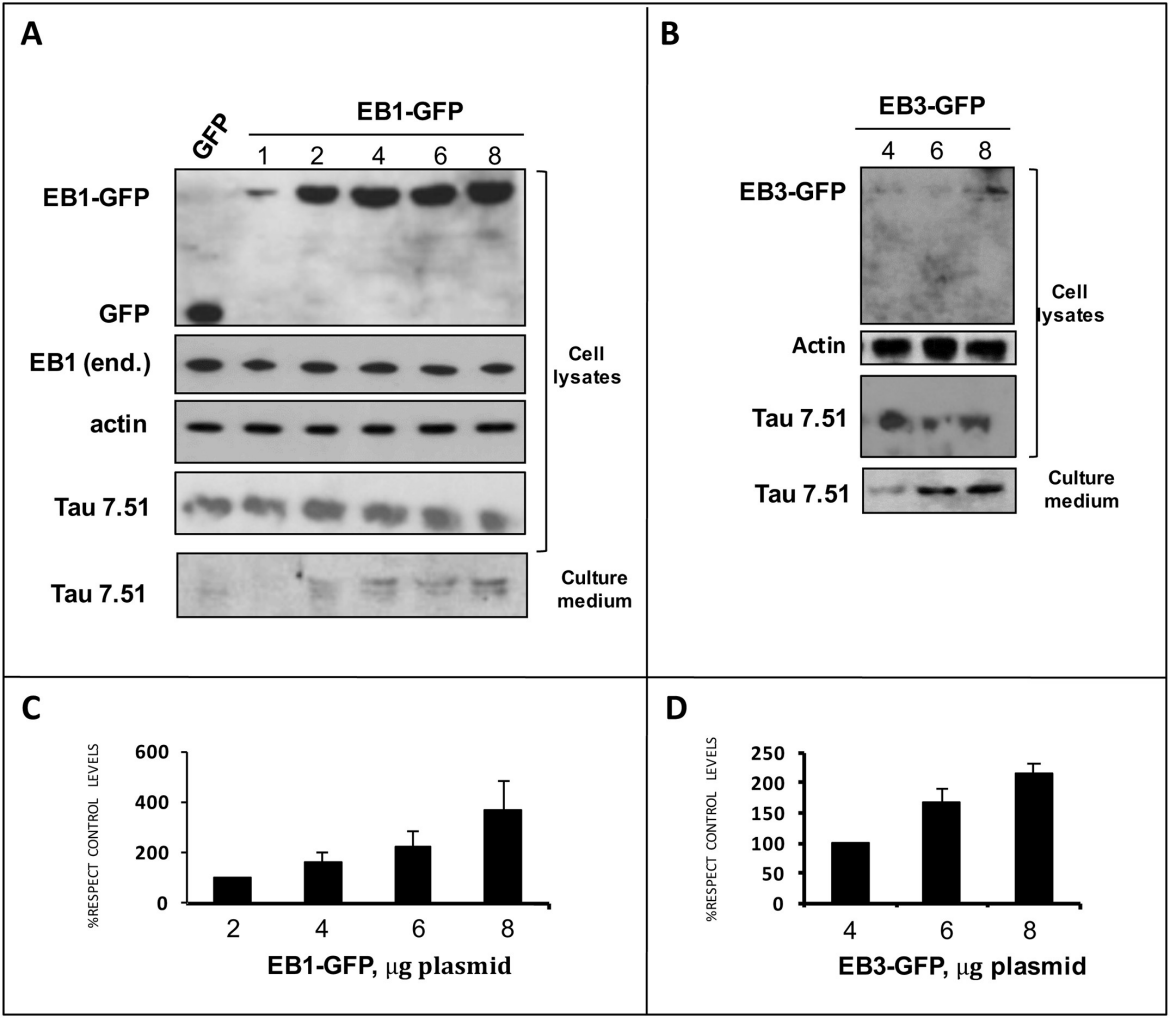
Overexpression of EB proteins facilitates tau secretion. A) Increasing amounts of EB1 protein were expressed in HEK-293-tau3R cells (μg plasmid per well). Increased EB1 expression levels (assessed with an anti-GFP antibody, upper panel) correlate with increased amounts of secreted tau observed in the culture medium (lower panel). Endogenous levels of EB proteins (EB1 end.) and intracellular tau remain unchanged. B) Similar results were obtained for EB3, although EB3 levels were much lower than of EB1, upon transfection (in line with this, endogenous levels of EB3 were barely detectable in these cells (data now shown)). A tau increase in the culture media was observed with an anti-tau antibody. No differences in intracellular tau levels were observed. C) Quantification of the data from A). D) Quantification of the data from B).

To test whether EB proteins may indeed play a role in tau secretion, as previously suggested for Interleukin-1ß secretion [14], we analyzed the effect of overexpressing EB proteins on tau release to the extracellular space (gain-of-function approach). GFP-tagged EB1 or EB3, or GFP alone as a control, were transfected in human HEK-293T cells stably expressing full length human tau (HEK-293-tau3R) and the extracellular tau present in the culture medium was detected by Western blot. We found a strong correlation between increasing amounts of overexpressed EB1 or EB3 proteins and an increase in full length human tau levels in the culture medium (Fig 4 and S1 Fig), suggesting that EBs might be involved in the secretion of tau protein to the extracellular milieu.

### Decreased EB protein expression impairs tau secretion

**Fig 5:**
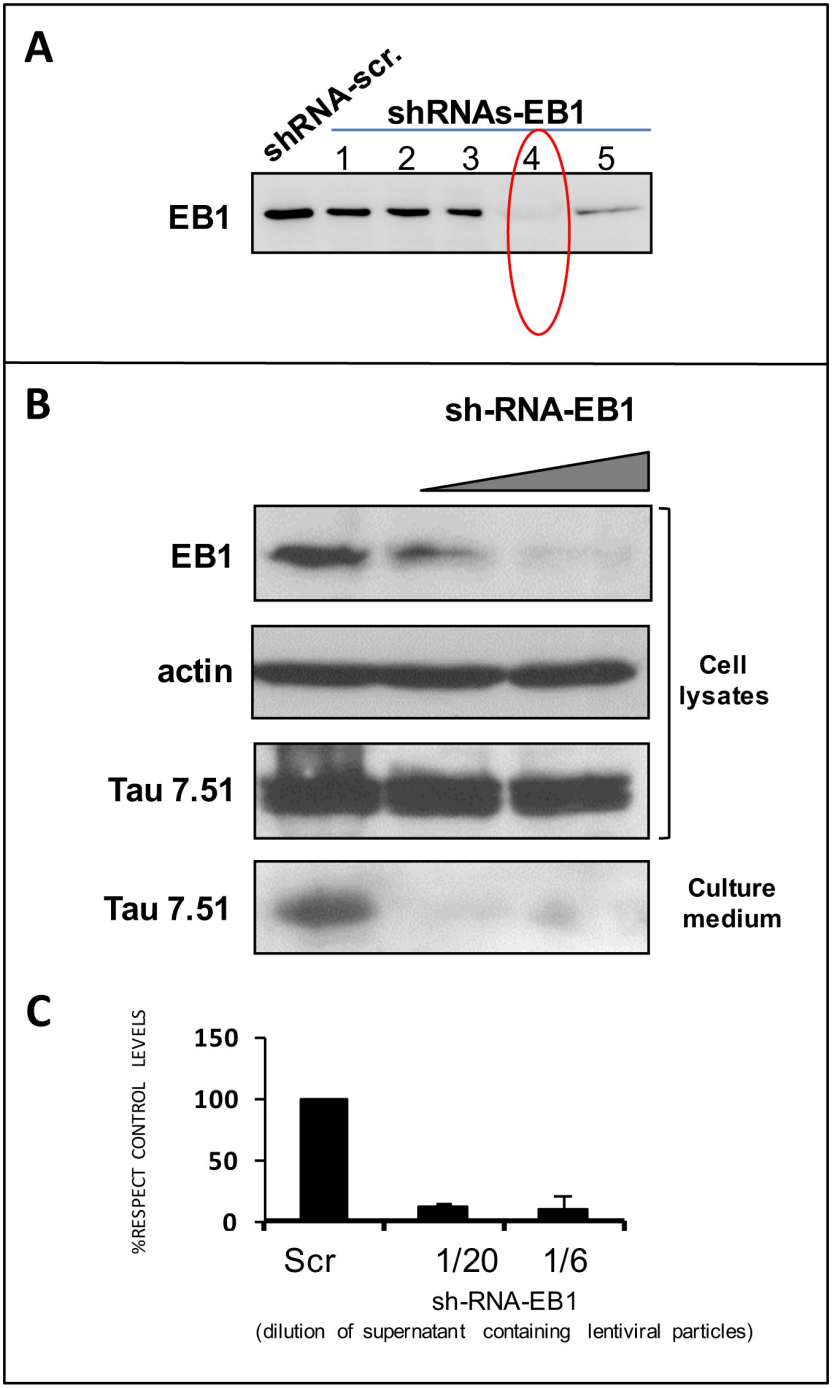
EB1 depletion decreases tau secretion. A) Different levels of EB1 downregulation upon transduction of five different specific shRNAs for human EB1 in HEK-293-tau3R cells. B) Increasing amounts of EB1-shRNA-4 lentiviral particles (dilution 1/20 and 1/6 of supernatant containing the lentiviral particles, see Methods section) were used to transduce HEK-293-tau3R cells, leading to a progressive decrease in endogenous EB1 levels (upper panel). This reduction of EB1 expression levels leads to a decrease amount of secreted tau in the culture medium (lower panel) without affecting the expression of intracellular tau. This reduction that not take place when scrambled shRNA (Src) was tested. C) Quantification of the data from B).

To confirm EBs participation in tau secretion, we decided to pursue a loss-of-function approach by downregulating the expression of EB proteins and looking for the effect on extracellular tau levels. Since endogenous EB3 was hardly detectable in our non-neuronal working cell model (data not shown), we focused our experiments on knocking down EB1. After testing five different EB1-speciic shRNAs, we selected the most effective one in depleting EB1 protein levels (shRNA number 4 in Fig 5A) to analyze its effect on the tau secretion process. As shown in Fig 5B and S2 Fig, transduction of HEK-293-tau3R cells with EB1 shRNA lentiviral particles leads to a significant decrease on tau levels in the culture medium. Overall, these data indicate that EB proteins modulate the release of tau proteins.

## Discussion

Tau is a microtubule-associated protein mainly expressed in neurons and mostly localized at the distal part of the axon. Tau protein is present in mammals such as mice or humans, although there are slight differences in its primary structure depending on the species [23]. Interestingly, those differences are preferentially located at the N-terminal domain of the tau molecule [24]. Despite of the small structural differences, tau in its monomeric form from different origins, is an unfolded, intrinsically disordered protein (IDP) [25]. This feature may allow the binding of different proteins, or molecules, to the exposed sites. One of those exposed sites, present in human but not in mouse tau is the motif spanning residues 18 to 28. To search for a specific function of this peptide, human tau (htau) or human “murinized” tau (htau-**Δ**18–28)–lacking this peptide- were overexpressed in non-neuronal cells. A higher secretion was found for the cells overexpressing htau as compared to those overexpressing htau-**Δ**18–28. In order to investigate a possible role of the htau peptide 16–28 in tau secretion, we looked for tau peptide-binding proteins by affinity chromatography and we present evidence that human tau peptide 16–28 interacts also with EB proteins -EB1 and EB3-, the core +TIPs. We had previously shown that tau interacts with EB proteins in neuronal cells and brain tissue [13]. This interaction was first suggested to occur through TXIP motifs present in tau (residues 95–98 and 169–172), similar to the canonical SXIP EB-interacting motif [26]. However, a recent report suggests that those residues are not involved in the binding of human tau to EB proteins; on the contrary, the microtubule-binding repeats of tau were shown to participate in its interaction with EBs [22]. Sequence analysis revealed some similarity between those repeats and the human tau 16–28 peptide sequence. Moreover, we showed here that tau 16–28 peptide binds to tubulin and actin, as shown before for tau microtubule-binding repeats [27], suggesting that both sequences could share some common function.

Since the htau peptide 16–28 interacts with EBs and this peptide seems to be involved in human tau secretion, we investigated the potential participation of EB proteins in tau secretion. Although EBs are key regulators of different aspects of neuronal biology, such as neurite/axon extension [21, 28], dendritic spine morphology [29] or axon initial segment regulation [30], EBs participation in protein secretion has not been much investigated in neurons. However, in non-neuronal cells, EBs have been reported to modulate secretion of some proteins involved in inflammation, such as interleukin-1 by regulating autophagy-dependent secretion [14]. Our results suggest that EB proteins could indeed favor tau secretion. In this way, we found that overexpression of EB proteins increases tau secretion whereas EB depletion impairs it in non-neuronal cells stably overexpressing tau. We recently showed that tau regulates the localization of EBs in the distal region of the axons [13], where tau is mostly present [10, 21]. Hence, there might be a crosstalk between tau and EBs in distal axons in neurons in which tau may contribute to proper EBs localization whereas EBs might facilitate tau release to the extracellular milieu.

It is widely known that intracellular tau accumulation could be toxic for cells. Previously, we have reported that an accumulation of intracellular tau results in the secretion of the protein [17] that could decrease the toxicity of intracellular tau. Now, we present evidence that the presence of peptide 18–28 in human tau could favor the secretion and propagation of tau molecule through its interaction with EB proteins. Thus, from our work, EBs emerge as potential regulators of tau release and spreading that occurs in tauopathies.

## Supporting information

S1 Table Shows information on human brain tissue samples used. (DOCX)

S1 Fig Overexpression of EB proteins (μg plasmid per well) facilitates tau secretion. Additional representative Western-blots for increased amounts of secreted tau observed in the culture medium of HEK-293-tau3R cells overexpressing EB1 (upper panel) and EB3 (lower panel) tested with anti-tau antibody 7.51.
(TIF)

S2 Fig EB1 depletion decreases tau secretion. Additional representative Western-blot for decrease amounts of secreted tau observed in the culture medium of HEK-293-tau3R cells infected with EB1-shRNA-4 lentiviral particles (dilution 1/20 and 1/6 of supernatant containing the lentiviral particles, see Methods section) tested with anti-tau antibody 7.51.
(TIF)
