# Supplementary material for: Role of tau N-terminal motif in the secretion of human tau by End Binding proteins
Source: PLoS One. 2019 Jan 22;14(1):e0210864. doi: 10.1371/journal.pone.0210864 (PMC6342323; doi:10.1371/journal.pone.0210864)
Supplement: S1 Table — (DOCX) [file pone.0210864.s001.docx]

**Supporting Table I. Table shows information on human brain tissue samples used.**

| GENDER | AGE | POST-MORTEM INTERVAL | BRAAK-TAU | Area |
| --- | --- | --- | --- | --- |
| MALE | 41 | 11.58h | 0 | FrontalCx |
| FEMALE | 54 | 8h | 0 | FrontalCx |
| FEMALE | 51 | 4h | 0 | FrontalCx |

Information regarding, gender, age, postmortem interval, Braak-Tau stage and cerebral area of samples used.
