# Supplementary figures and images for: Role of tau N-terminal motif in the secretion of human tau by End Binding proteins
Source: PLoS One. 2019 Jan 22;14(1):e0210864. doi: 10.1371/journal.pone.0210864 (PMC6342323; doi:10.1371/journal.pone.0210864)

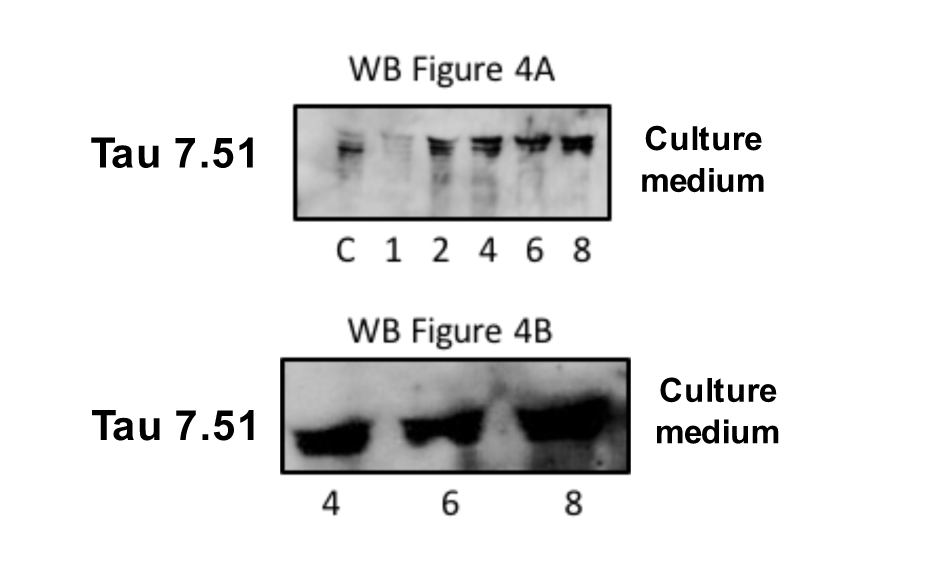

Supplement: S1 Fig — Additional representative Western-blots for increased amounts of secreted tau observed in the culture medium of HEK-293-tau3R cells overexpressing EB1 (upper panel) and EB3 (lower panel) tested with anti-tau antibody 7.51. (TIF) [file pone.0210864.s002.tif]

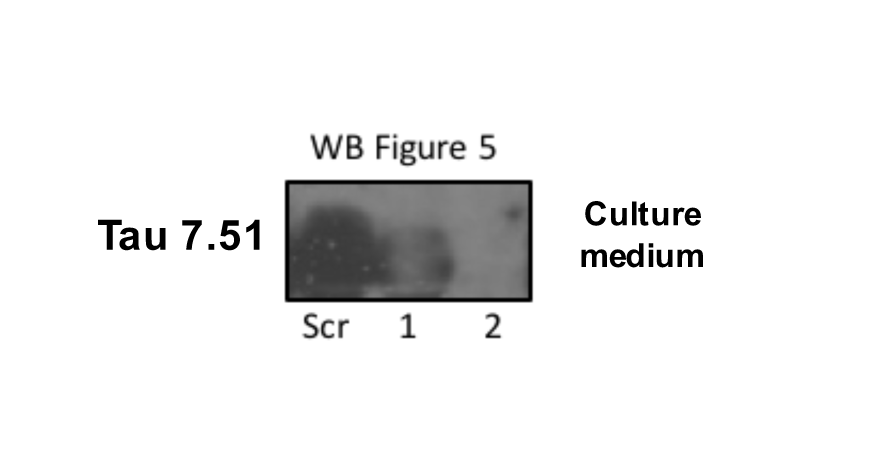

Supplement: S2 Fig — Additional representative Western-blot for decrease amounts of secreted tau observed in the culture medium of HEK-293-tau3R cells infected with EB1-shRNA-4 lentiviral particles (dilution 1/20 and 1/6 of supernatant containing the lentiviral particles, see Methods section) tested with anti-tau antibody 7.51. (TIF) [file pone.0210864.s003.tif]
